# Supplementary material for: Transmission matrix parameter estimation of COVID-19 evolution with age compartments using ensemble-based data assimilation
Source: PLoS One. 2025 Apr 28;20(4):e0318426. doi: 10.1371/journal.pone.0318426 (PMC12036932; doi:10.1371/journal.pone.0318426)
Supplement: Attachment — Submitted filename: review.pdf [file pone.0318426.s001.pdf]

Dear editor,

The manuscript entitled **"Transmission matrix parameter estimation of COVID-19 evolution with age compartments using ensemble-based data assimilation "** presents an stochastic compartimental epidemiological model with Susceptible-Exposed-Infective-Mild-T(severe)-Critical-Recovered-Deceased compartments for the Covid-19 spread in Argentina. In addition to that, based on Kalman filter techniques, the model allows for time-dependent parameters. This model is very complex with many state variables (24 in total) and 18 parameters. In addition to the deterministic time evolution of the dynamical system compartimental model, they add noise to the data, which comes from subnotification of cases, delays in case reporting, etc that can be classified as observational errors. The mathematical tools used by the authors seem to be a relevant application to the field of epidemiological modeling.
However, there are issues in the manuscript (MS) that the authors should address before the MS could be accepted for publication in the PLOS ONE. Most of these issues are minor and I believe the authors could easily fix them to make the MS more readable and accessible to a wider readership. Below I list the identified issues point by point.

1 Main issues

1. It seems to be a very daunting problem to keep track of all these state variables and parameters. The authors should emphasize the advantage of their model over existent simpler epidemiological models with time-dependent parameters.

2. It is not very clear from the text how all the parameters of the model are updated to better fit the available epidemiological data. Where is the input of epidemiological data in the Algorithm 1?

3. The compartment diagram of Fig. 1 shows the basic structure of the dynamics of the model. Why no vertical transitions are allowed between the M_j, T_j and C_j compartments. Symptoms may start mildly and evolve to T_j and C_j. The converse is also possible, they may reverse from C_j to M_j before recovery.

4. It is not clear what the authors mean by "incident cases" in Fig. 3 and elsewhere in text and in other figures. Incident cases of what? Do they mean confirmed cases? infective cases? Something else?

5. I am not sure how the authors extracted the data they used from the Argentinian government site (datos.salud.gob.ar/dataset/covid-19-casos-registrados-en-la-republica-argentina). There are several huge csv files in there. I recommend the authors discuss in more details, perhaps in a supplementary material, what data they used and how they extracted the necessary information from these files.

6. In Fig. 3, what the authors mean by true and obs data? Is the obs data the epidemiological data found in the provided site above?

7. In Fig. 7, I can only see the obs and IC3 data. Where are the other results for IC 1 and IC 2? Also, which compartments of the model the ICs represent?

8. How could this model be applied or adapted to other populations (cities, countries, regions, etc)?

9. I believe in many populations (cities, states or countries) around the world not as much information is available about the spread of Covid, such as the segmentation of the data by age, gender, gravity of the disease, discharge date from hospital, etc. How could this model be adapted to simpler situations, with less available data, such as was studied in Ref. [1], where an automatic time dependent parameter estimation was implemented based on the available public data.

10. Other types of modeling and forecasting available in the literature are Monte Carlo Markov-Chain models for the spread of Covid and for forecasting such as [1, 2, 3, 4]. How the proposed model of parameter estimation could improve upon the forecasting abilities of these existing models?

11. During the early stages of the pandemics spread there should be more fluctuations due to the small number of infected people then. This probably impacts the predictive ability of the model. How did the authors circumvented this type of problem?

12. Usually, R_{eff} is higher right at the beginning of the spread of the pandemics, when there was no lockdown, people were not using masks, etc, but in Fig. 10 the peak in R_{eff} came a month later approximately. This result seems counterintuitive.

13. What exactly do the authors mean with the sentence on page 3, lines 88-89: *"The evolution of epidemiological parameters is estimated over a long time period assuming a prior density for them."* What prior density is this? It is not clear from the text at this point (pg. 3 ln. 89). Only in pg. 7 the authors discuss about a conditional probability density function, but for state variables not for parameters. Again, in pg. 12 lines 389-391 the authors say *"To examine the identifiability and sensitivity to initial conditions of the estimated parameters, three experiments with different apriori density of parameters at $t = 0$ are shown."*. I found nowhere in the text of the MS any equation for the evolution of a probability density function (pdf) of the parameters. Eq. (14) is related to the stochastic evolution of the parameters themselves, not to a pdf.

14. The following issue is related to the previous one. On the last paragraph of page 13, the authors mention *"The three experiments converge ..."*. What experiments are these? I would naively think that 3 sets of parameters were generated from the same pdf at $t = 0$.

15. Further down, on the last paragraph of page 14, the authors write *"Contrary to the twin experiments ..."*. What twin experiments are these?

16. Equation (2) is analogous to the principle of detailed balance that occurs in equilibrium statistical mechanics systems and in reversible Markov chains. This implies that entropy increases over time in isolated physical systems. The authors could comment on this if they find the concept of entropy relevant for epidemiology.

17. What is the difference between model errors η_k in eq (5) and observation errors ϵ_k in eq (6)? What are their causes?

18. What is the pdf that generates the noise ϵ_k?

19. Why 24 state variables? I would expect 21 due to the 3 constraints for N_j, which are fixed numbers.

20. The authors should include citation(s) backing and/or comment on the choice of parameter values given in Table 2. I think some of them might be obtained from the epidemiological data.

2 Minor issues

2.1 Typographical and syntactic errors

1. "widespread of SARS-COV-2"→ spread of SARS-COV-2

2. may help to prevent → may help prevent

3. disagregated, perhaps a better alternative is segmented

4. intensive cares → intensive care

5. dependent of → dependent on

6. days after → 'days later' or 'days afterwards'

7. Just after 16 days of this → Just 16 days later,

8. In the previous shown → In the previously shown

9. consist of → consist on

10. model parameters has → model parameters have

11. transmission matrix 15 → transmission matrix (15)

12. between young and adults → between young people and adults

13. transmission matrix 16 → transmission matrix (16)
14. 15 day lead ... → 15-day lead ...
15. are then extrapolate → are then extrapolated
16. forecast accurate → forecast accuracy
17. agegroup → age group
18. In table I, sever → severe
19. by the constrain → by the constraint

2.2 Figures

1. I suggest the figures be limited to the confines of the data available. The horizontal axes should start with the first data point and end with the last data point.
2. The plots seem rather fuzzy. Perhaps they are jpeg files. I recommend the authors use instead pdf figures, if PLOS ONE accepts them, or higher definition figures.
3. The authors should comment on why there are discontinuities in the forecast curve of Fig 11. In some cases widths of a different color appear, but do not seem to be discussed in the text.
4. What is the meaning of q, I and c labels at the bottom of Fig 12?

2.3 Acronyms

Please spell out the first appearance of acronyms in the text such as: ABM, NGO, and RMSE. Also, to avoid ambiguity, the authors should include the mathematical formula of RMSE, since it might mean different things in different papers.

3 Code availability

I recommend the authors include the code as supplementary material or include a link to a repository such as GitHub. Definitely, more details about the algorithm used should be given, since the techniques used are not as well known in epidemiological modeling as they are in weather forecasting.

4 Conclusion

This MS applies a set of statistical techniques, usually used in weather forecasting, to the modeling and forecasting of the spread of Covid. These techniques seem to be powerful and well tested in other fields. I think its publication by the PLOS ONE will be beneficial to the scientific community if the authors address the issues raised in this report so as to make the MS more readable to a wider readership.

References

[1] Adriano A Batista and Severino Horácio da Silva. "An epidemiological compartmental model with automated parameter estimation and forecasting of the spread of COVID-19 with analysis of data from Germany and Brazil". In: *Frontiers in Applied Mathematics and Statistics* 8 (2022), p. 645614. DOI: 10.3389/fams.2022.645614.

[2] Andrea L. Bertozzi et al. "The challenges of modeling and forecasting the spread of COVID-19". In: *Proceedings of the National Academy of Sciences* 117.29 (2020), pp. 16732–16738. DOI: 10.1073/pnas.2006520117. URL: https://www.pnas.org/content/117/29/16732.

[3] Y Fang, Y Nie, and M Penny. "Transmission dynamics of the COVID-19 outbreak and effectiveness of government interventions: A data-driven analysis". In: *Journal of Medical Virology* 92 (2020), pp. 645–659. DOI: 10.1002/jmv.25750.

[4] Jonas Dehning et al. "Inferring change points in the spread of COVID-19 reveals the effectiveness of interventions". In: *Science* 369 (2020). DOI: 10.1126/science.abb9789.
