# Supplementary material for: Transmission matrix parameter estimation of COVID-19 evolution with age compartments using ensemble-based data assimilation
Source: PLoS One. 2025 Apr 28;20(4):e0318426. doi: 10.1371/journal.pone.0318426 (PMC12036932; doi:10.1371/journal.pone.0318426)
Supplement: Attachment — Submitted filename: responses.pdf [file pone.0318426.s002.pdf]

## Responses to the review

## "Transmission matrix parameter estimation of age-disaggregated COVID-19 evolution using ensemble-based data assimilation"

by S. Rosa et al.

We would like to thank the reviewer for the careful read of the manuscript. We believe his/her constructive comments and precise suggestions helped to improve substantially this manuscript.

We state the reviewer comments and then answers to his/her comments follow.

1. *It seems to be a very daunting problem to keep track of all these state variables and parameters. The authors should emphasize the advantage of their model over existent simpler epidemiological models with time-dependent parameters.*

   We have now added some sentences to emphasize the advantages of our age-disagregated model in the introduction line 85 and in the conclusions, see lines 555 and 575. We have also tried to emphasize in the manuscript the importance of employing techniques for real time monitoring using age-disaggregated data, which is crucial for an effective response to epidemics, as evidenced by numerous government statements and publications (Diaz et al. 2021; Davis et al 2020, Heidari et al 2020). The reasons and motivations for age-disagregated techniques including the aforementioned references are now stated in line 64.

2. *It is not very clear from the text how all the parameters of the model are updated to better fit the available epidemiological data. Where is the input of epidemiological data in the Algorithm 1?*

   Following the reviewer comment, we updated the description of some paragraphs of section 2.2. In particular, we added the statement "Data input as perturbed observations" in Algorithm 1.

3. *The compartment diagram of Fig. 1 shows the basic structure of the dynamics of the model. Why no vertical transitions are allowed between the $M_j$, $T_j$ and $C_j$ compartments. Symptoms may start mildly and evolve to $T_j$ and $C_j$. The converse is also possible, they may reverse from $C_j$ to $M_j$ before recovery.*

   We now clarify the meaning of these categories in line 119.

4. *It is not clear what the authors mean by "incident cases" in Fig. 3 and elsewhere in text and in other figures. Incident cases of what? Do they mean confirmed cases? infective cases? Something else?*

   We referred to 'incident cases' as the new cases appearing everyday. We now call them 'daily cases' following Li et al. to clarify the manuscript. We define and justify daily cases in line 403.

5. *I am not sure how the authors extracted the data they used from the Argentinian government site (datos.salud.gob.ar/dataset/covid-19-casos-registrados-en-la-republica-argentina). There are several huge csv files in there. I recommend the authors discuss in more details, perhaps in a supplementary material, what data they used and how they extracted the necessary information from these files.*

   We uploaded our curated data to a public repository. The link to the repository appears in the new version of the manuscript. We will make available the codes used to download and process the data after the manuscript is acepted. This was added in a new section about Code and data availability, line 619.

6. *In Fig. 3, what the authors mean by true and obs data? Is the obs data the epidemiological data found in the provided site above?*

   In line 290 we now clarify how synthetic observations are produced. We also state more clearly this distinction in the figure captions.

7. *In Fig. 7, I can only see the obs and IC3 data. Where are the other results for IC 1 and IC 2? Also, which compartments of the model the ICs represent*

   'IC1', 'IC2' and 'IC3' stands for 'initial condition' 1, 2 and 3 respectively. We now define it and then explicitly say it in each figure caption mentioning them.

8. *How could this model be applied or adapted to other populations (cities, countries, regions, etc)?*

   The EnKF makes no specific assumption about the observations. In fact, we use two types of observation: synthetic and real data. In terms of the epidemiological model only the age-disagragated population needs to be specified (under the assumption that the population may be considered isolated in the mean field approximation). We have added the point asked for the reviewer in Line 587.

9. *I believe in many populations (cities, states or countries) around the world not as much information is available about the spread of Covid, such as the segmentation of the data by age, gender, gravity of the disease, discharge date from hospital, etc. How could this model be adapted to simpler situations, with less available data, such as was studied in Ref. [1], where an automatic time dependent parameter estimation was implemented based on the available public data.*

   Actually, the aim of the work was to show the impact of having the information segmented by ages. As mentioned by the reviewer in simpler situations there are some works that show parameter estimation. The work compares the forecast resulting from the age-group disaggregation and without it (well-mixed model with time dependent parameter estimation) showing the added value of the segmented assimilation of data. As mentioned, we now emphasize this point in Line 575. The importance of age-disaggregated data has been underlined by Diaz et al 2021; Heidari et al 2020 as a prerequisite for an effective response to epidemics. We agree with the reviewer that for many countries age-disaggregated information was not available

but we believe it is important to show them the added value of this data. Textually from Heidari et al. "Countries have an obligation to share comprehensive and accurate data disaggregated by sex and age as a minimum (as requested in WHO aggregate reporting) ... for better understanding of the evolving pandemic".

We have also added the reference suggested by the reviewer in Line 40.

10. *Other types of modeling and forecasting available in the literature are Monte Carlo Markov-Chain models for the spread of Covid and for forecasting such as [1, 2, 3, 4]. How the proposed model of parameter estimation could improve upon the forecasting abilities of these existing models?*

    We added the pertinent references to the introduction in line 40. There is a work comparing MCMC modelling and data assimilation inference. This reference was added in line 489.

11. *During the early stages of the pandemics spread there should be more fluctuations due to the small number of infected people then. This probably impacts the predictive ability of the model. How did the authors circumvented this type of problem?*

    We are aware of this problem. In fact, the EnKF works smoothly for mean-field models with a relatively large number of cases. We point this out in the line 535.

12. *Usually, $R_{eff}$ is higher right at the beginning of the spread of the pandemics, when there was no lockdown, people were not using masks, etc, but in Fig. 10 the peak in $R_{eff}$ came a month later approximately. This result seems counterintuitive.*

    Good point. The prior mean parameters for $R_{\text{eff}}$ that we were using were smaller than the estimated values for the early state of the pandemic and so not very representative.We conducted new experiments adding a case with a larger prior mean reproduction number $R_{\text{eff}}$ and commented the interesting results of these experiments in line 497.

13. *What exactly do the authors mean with the sentence on page 3, lines 88-89:"The evolution of epidemiological parameters is estimated over a long time period assuming a prior density for them." What prior density is this? It is not clear from the text at this point (pg. 3 ln. 89). Only in pg. 7 the authors discuss about a conditional probability density function, but for state variables not for parameters. Again, in pg. 12 lines 389-391 the authors say "To examine the identifiability and sensitivity to initial conditions of the estimated parameters, three experiments with different apriori density of parameters at t =0 are shown.". I found nowhere in the text of the MS any equation for the evolution of a probability density function (pdf) of the parameters. Eq. (14) is related to the stochastic evolution of the parameters themselves, not to a pdf.*

    We take Gaussian prior densities for the parameters as for the state variables. We have added a clarification about this point in line 231. In general to assess identifiability of the parameters we conducted different experiments in which the mean of the prior density at $t = 0$ has different values (explained in next point 14).

14. *The following issue is related to the previous one. On the last paragraph of page 13, the authors mention "The three experiments converge ...". What experiments are these? I would naively think that 3 sets of parameters were generated from the same pdf at $t = 0$.*

    The three experiments are representing three different prior densities for the parameters. We set three different mean parameters at the initial time. A definition of the term IC1, IC2 and IC3 was lacking in the previous manuscript. This is now clarified in the corrected manuscript in line 387.

15. *Further down, on the last paragraph of page 14, the authors write "Contrary to the twin experiments ...". What twin experiments are these?*

    We realised that we were using the term without defining it. We have now added a paragraph in line 403 where we define the meaning of twin experiments. We now try to use, instead of twin experiments, the term synthetic observation experiments, which we think is more representative for a general audience.

16. *Equation (2) is analogous to the principle of detailed balance that occurs in equilibrium statistical mechanics systems and in reversible Markov chains. This implies that entropy increases over time in isolated physical systems. The authors could comment on this if they find the concept of entropy relevant for epidemiology.*

    We are not familiar with this topic. The topic sounds interesting and we apreciate further comments by the reviewer and a reference. We have added to the manuscript a reference of Cappe's book on inference in hidden Markov models (line 170). In this reference Equation (2) is discussed in a broader context.

17. *What is the difference between model errors $\eta_k$ in eq (5) and observation errors $\epsilon_k$ in eq (6)? What are their causes?*

    We now mention in line 172 about the source of model errors.

18. *What is the pdf that generates the noise $\epsilon_k$?*

    Observational errors are assumed Gaussian white noise with mean $\mathbf{0}$ and covariance matrix $\mathbf{R}$. We added this in line 172 and 181.

19. *Why 24 state variables? I would expect 21 due to the 3 constraints for Nj, which are fixed numbers.*

    We clarify now that there are only 21 independent variables due to the constraints (line 359).

20. *The authors should include citation(s) backing and/or comment on the choice of parameter values given in Table 2. I think some of them might be obtained from the epidemiological data.*

    We now added references for the choice of parameter values we use in line 140.

## Comments on the figures

1. *I suggest the figures be limited to the confines of the data available. The horizontal axes should start with the first data point and end with the last data point.*

   We implemented this suggestion.

2. *The plots seem rather fuzzy. Perhaps they are jpeg files. I recommend the authors use instead pdf figures, if PLOS ONE accepts them, or higher definition figures.*

   We increased the image resolution and used a vectorized figure format (eps). We expect this issue is solved now.

3. *The authors should comment on why there are discontinuities in the forecast curve of Fig 11. In some cases widths of a different color appear, but do not seem to be discussed in the text.*

   We comment about this plot in line 529.

4. *What is the meaning of q, I and c labels at the bottom of Fig 12?*

   We added the meaning of q, l and c in the caption of Fig 12.

*Acronyms* We define the acronyms ABM and RMSE including the used formula of RMSE. The acronym NGO was removed from the text.
*Code availability* We added a short section about the code and data source availability statement with the corresponding links at the end of the manuscript.

We thank the reviewer for all the pertinent comments about the typographical and grammar errors. We would like to express our gratitude to the reviewer for conducting a thorough review of the manuscript and for providing us with all the relevant comments.

## References

Davies, N.G., Klepac, P., Liu, Y., Prem, K., Jit, M. and Eggo, R.M., 2020. Age-dependent effects in the transmission and control of COVID-19 epidemics. Nature medicine, 26(8), pp.1205-1211.

Diaz, T., Strong, K.L., Cao, B., Guthold, R., Moran, A.C., Moller, A.B., Requejo, J., Sadana, R., Thiyagarajan, J.A., Adebayo, E. and Akwara, E., 2021. A call for standardised age-disaggregated health data. The Lancet Healthy Longevity, 2(7), pp.e436-e443.

Heidari S, Ahumada C, Kurbanova Z, Gender GE., 2020. Towards the real-time inclusion of sex-and age-disaggregated data in pandemic responses. BMJ global health. 5(10).
