# Supplementary material for: Transmission matrix parameter estimation of COVID-19 evolution with age compartments using ensemble-based data assimilation
Source: PLoS One. 2025 Apr 28;20(4):e0318426. doi: 10.1371/journal.pone.0318426 (PMC12036932; doi:10.1371/journal.pone.0318426)
Supplement: Attachment — Submitted filename: responses_auresp_2.pdf [file pone.0318426.s003.pdf]

Responses to the review

"Transmission matrix parameter estimation of COVID-19 evolution with age compartments using ensemble-based data assimilation"

by S. Rosa et al.

We would like to thank the reviewer again for the careful read of the second version of the manuscript. We believe his/her suggestions helped us to improve substantially the manuscript. We state the reviewer comments and then answers to his/her comments follow.

1. *The authors should cite and/or include in the text the equations of motion of the well-mixed SEIR model mentioned in line 542. This could be in the supplementary material. I believe the parameters could be obtained from Model (1) and Table 1. The parameters of this simplified model should be informed as well. Are the parameters also time dependent? I think the same type of algorithm is used in both systems (with the meta-population model and the well-mixed SEIR model)*

   We now state in line 561 that the well-mixed model is obtained from Model (1) setting $n = 1$.

2. *The time-step evolution operator of the dynamical model M can be obtained clearly from equation (1). It is still not very clear which parameters constitute the parameter vector $\boldsymbol{\theta}_k$ in the stochastic eq. (12). From the figures, it seems the infection coefficients, the gammas, and the fractions f_j^C, with $j = 1, 2, 3$, jointly constitute the vector $\boldsymbol{\theta}_k$. What the vector $\boldsymbol{\theta}_k$ is exactly should be stated in the manuscript.*

   In line 242 we now state that the vector $\boldsymbol{\theta}_k$ represent the parameters to be estimated. Additionally, in lines 380 and 383 we show the form of $\boldsymbol{\theta}_k$ in the twin experiments, and in the line 480, the form of $\boldsymbol{\theta}_k$ in the real data experiments.

3. *How is the observational covariance matrix R just below eq.(4) obtained?*

   The observation covariance is not estimated, its variances (diagonal terms) are prescribed according to the observation error of the involved variables. This is discussed in line 191, and then variances are specified in line 346. Also we added a pertinent reference [1] about the estimation of the covariance matrix in line 192.

4. *To make the manuscript more readable, just below eq. (7), I suggest explaining what the forecast step means, perhaps referencing Algorithm 1.*

   In the line 212 We now state what the forecast step is.

5. *I think the authors should include the step in which $\mathbf{y}_k$ is obtained in the Algorithm 1 description.*

In Algorithm 1 is stated that $\mathbf{y}_k$ is required as an input. We noted a typo the previous version: the observations were displayed with the uppercase letter $\mathbf{Y}_k$, which has now been corrected to $\mathbf{y}_k$.

6. *The authors could refer to https://en.wikipedia.org/wiki/Detailed_balance for more information on the detailed balance importance to Statistical Physics.*

   We have now included a sentence commenting on detailed balance (line 195) in the context of a hidden Markov model.

7. *The authors could inform the reader what is the specific matrix H used just below equation (11).*

   In line 237 we now state what the matrix $\mathbf{H}$ is.

8. *When printed in black and white it is hard to see the differences of the darker solid/dashed lines. This is seen most notably in figs. 2, 4, 8, 10, 12. I recommend the authors use different thicknesses or line styles in addition to color so as to better distinguish them.*

   We updated all plots with different line styles and changed the caption of the Fig 12.

9. *In the end of the Introduction section, I think the authors inadvertently erased the paragraph with the short description of the remaining sections of the manuscript.*

   In line 96 we added an outline of the article.

10. *I did not find the added reference comparing MCMC models and data assimilation inference in line 489.*

    There was a mistake in the line reference. We corrected the number to line 513 and added a comment on that Sauer et al. (2021) compared MCMC and data assimilation methods.

References

1. Pulido M, Tandeo P, Bocquet M, Carrassi A, Lucini M. Stochastic parameterization identification using ensemble Kalman filtering combined with maximum likelihood methods. Tellus A: Dyn Met and Oc. 2018;70(1): 117. https://doi.org/10.1080/16000870.2018.1442099
2. Sauer T, Berry T, Ebeigbe D, Norton MM, Whalen A, Schiff S. Identifiablility of infection model parameters early in an epidemic. Siam J Control Optim. 2022;60(2): S27S48. https://doi.org/10.1137/20M1353289.
